# A M.O.H. On Boots or No Boots

**Published:** 1918-09-28

**Authors:** R. King Brown

**Affiliations:** Medical Officer of Health. Public Health Department, Town Hall, Bermondsey, S.E. 16.


*To the Editor of* The Hospital.

### A M.O.H. on Boots or No Boots.

*To the Editor of* The Hospital.

Sir,—I was making inquiries recently at one of my Maternity and Child-Welfare Centres on the expenses which poor mothers have to face in the weekly budget, and I found that if they have many children the question of boots is all-important. One mother, who had six children, informed me that soleing and heeling of the smallest pair of boots now costs at least 2s. instead of 9d. or 10d. pre-war, and that the price varies from this up to 3s. and more, according to the size. The price of the smallest pair in this district is 7s. 11d. and the largest 13s. 11d., and they are of very poor quality. One pair she had went to bits in twenty-four hours, on account of the cardboard and paper stiffening. I have long thought that no boots at all are better, from a health point of view, than leaky ones. If a child goes to school unshod, his feet at least get dry and warm as soon as he gets into school, whereas if he has defective boots his feet remain wet and cold the whole day long, and it is quite obvious that this is very detrimental to health, especially in winter.

As the expense of repairing is now so great, would it not be possible for either the local authorities or some philanthropic committee formed for the purpose to provide a centre where poor people could have boots properly repaired at a reasonable price? If something could be done in this direction, I believe it would be an inestimable boon to many poor mothers with large families, and would leave them more money to spend on the necessities of life.—Yours, etc.,

R. King Brown, Medical Officer of Health.
Public Health Department, Town Hall,
Bermondsey, S.E. 16., September 17, 1918.

